# Characteristics of Evoked Potential Multiple EEG Recordings in Patients with Chronic Pain by Means of Parallel Factor Analysis

**DOI:** 10.1155/2012/279560

**Published:** 2012-02-02

**Authors:** Juan Wang, Xiaoli Li, Chengbiao Lu, Logan J. Voss, John P. M. Barnard, Jamie W. Sleigh

**Affiliations:** ^1^Institute of Electrical Engineering, Yanshan University, Qinhuangdao 066004, China; ^2^National Key Laboratory of Cognitive Neuroscience and Learning, Beijing Normal University, Beijing 10088, China; ^3^Department of Anesthesia, Waikato Hospital, Hamilton 3204, New Zealand

## Abstract

This paper presents an alternative method, called as parallel factor analysis (PARAFAC) with a continuous wavelet transform, to analyze of brain activity in patients with chronic pain in the time-frequency-channel domain and quantifies differences between chronic pain patients and controls in these domains. The event related multiple EEG recordings of the chronic pain patients and non-pain controls with somatosensory stimuli (pain, random pain, touch, random touch) are analyzed. Multiple linear regression (MLR) is applied to describe the effects of aging on the frequency response differences between patients and controls. The results show that the somatosensory cortical responses occurred around 250 ms in both groups. In the frequency domain, the neural response frequency in the pain group (around 4 Hz) was less than that in the control group (around 5.5 Hz) under the somatosensory stimuli. In the channel domain, cortical activation was predominant in the frontal region for the chronic pain group and in the central region for controls. The indices of active ratios were statistical significant between the two groups in the frontal and central regions. These findings demonstrate that the PARAFAC is an interesting method to understanding the pathophysiological characteristics of chronic pain.

## 1. Introduction

Chronic pain is a complex disease characterized by pain persisting after damage or pathology has healed. Effective treatment of chronic pain is hampered by an incomplete understanding of the pathophysiological changes that occur in the nervous system of chronic pain sufferers. The electroencephalogram (EEG) records the electrical activity from the scalp produced by the firing of neurons within the cerebral cortex [1] and has been widely used to analyze neural activity in chronic pain subjects [2, 3].

To investigate the physiological basis of chronic pain, event-related potentials (ERPs) have been used to explore pain-related modulation of the latency, location, amplitude, and frequency of evoked EEG responses to sensory stimulation [4–6]. Previous studies have shown that frequency and time domain characteristics of EEG recordings from certain brain regions are altered with chronic pain [7–9]. Traditionally, the ERP components are analyzed at specific cortical locations, that is, the vertex and frontotemporal region (e.g., [10, 11]). With this approach, however, the information provided is limited to the particular region under investigation and neglects the importance of wider cortical regions in information processing [12].

Multiple EEG recordings can be used to characterise the electrical activity across the whole cortex. Traditional methods of PCA and ICA analysis process the multiple EEG signals in two-way domains such as time channel and frequency channel [13]. To effectively characterise multiple EEG signals, development of an analysis method that captures time-frequency-channel information is required.

In this study, we have investigated differences in multiple ERPs between chronic pain patients and pain-free individuals. To characterise the EEG signals in the time-frequency-channel domain, a parallel factor analysis (PARAFAC) method with wavelet transforms was developed to decompose the multiple EEG recordings. The PARAFAC method has been successfully employed to detect abnormal EEG activity in neurological diseases such as epilepsy and Alzheimer's disease [14]. Herein, this novel method is employed to analyze the EEG in chronic pain subjects. The results show differences in cortical evoked activity between chronic pain and pain-free individuals and indicate that the PARAFAC is an effective method for extracting the characteristics of multiple EEG recordings in the time-frequency-channel domain.

## 2. Materials and Methods

### 2.1. Subjects

Subjects were 13 chronic pain patients recruited through the Waikato Hospital Pain Clinic and 13 pain-free volunteers. A range of conditions were represented in the patient group, including chronic lower back pain, neck pain, abdominal pain, and throat pain. None of the subjects in either group had a history of other neurological disease or head injury. Ethical approval was obtained from the Waikato Ethics Committee, and all subjects signed written informed consent.

### 2.2. Sensory Stimulations

The stimuli consisted of brief (10 ms), repetitive (at least 120) electrical shocks delivered to the dominant index finger. The electrodes were positioned on the dorsal aspect of the distal interphalangeal joint and a fine wire with a soldered tip, applied to the pulp of the fingertip. The stimulus intensity was recorded as the percent maximum voltage and rated by each subject on a 1–10 analogue scale. Two intensities of electric shock were tailored to each subject, one that was easily felt but not painful (from here on referred to as the “touch” stimulus) and one that was rated as “moderately painful” (the “pain” stimulus). The pain stimulus was felt as a sharp pricking sensation, predominantly under the wire electrode on the finger pulp. The shocks were given in three sequences at a constant frequency of 1 every 1.5 s as follows: (1) 120 sequential touch shocks (“touch”); (2) 120 sequential painful shocks (“pain”); (3) a random sequence of 300 touch and painful shocks at a 4 : 1 ratio. For the randomized protocol, the touch and pain stimuli were analyzed separately (“random touch” and “random pain”, resp.). Stimulus intensity and delivery were controlled by MatLab software (Matlab 6.0 Mathworks, Natick, MA, USA) running on a laptop computer that interfaced directly with the stimulus generator.

### 2.3. EEG Recording and Experimental Protocol

The subjects were comfortably seated, and the stimulating and EEG recording electrodes were attached. The latter consisted of a 28-channel bipolar montage configured in accordance with the international 10 : 20 system. The electrodes were Ag/AgCl sintered ring electrodes (Falk Minow, Herrsching, Germany) (1 cm outer diameter) that fastened securely to plastic loops imbedded in a prefabricated scalp cap (Easycap, Falk Minow, Herrsching, Germany). One of two cap sizes was chosen to give the correct positioning of the electrodes on the head relative to the nasion and inion, in accordance with the international 10 : 20 system. The centres of the electrodes were filled with an electrolyte gel, and attention was given to ensure the gel made contact with the scalp. Two reference electrodes were positioned behind each ear. The EEG electrodes were connected to two 16-channel biosignal amplifiers (Guger Technologies, Herbersteinstrasse, Austria) and digitised (Gdaqsys, Guger Technologies, Herbersteinstrasse, Austria) to computer at 100 Hz for continuous display and later offline analysis. The amplifiers were powered using mains-charged battery packs. One of the spare channels on the amplifier was used as an event marker from the electrical stimulus generator. Application of the electrodes took approximately 1 hour. The quality of the EEG was assessed by visual inspection and corrective measures taken to improve the quality of “noisy” channels. This usually involved checking the contact of the electrolyte gel between the electrode and the scalp. Time restraints, particularly with the requirement for patients to be seated for up to two hours to complete the study, meant it was not practicable to check and monitor individual channel impedances.

During delivery of the stimulation sequences, the subjects were instructed to keep their eyes closed, refrain from talking, and relax as much as possible. The subjects were not specifically instructed to either attend to or ignore the stimuli. The subjects could stop the stimulation at any time by pressing a button. The three sequences took approximately 30 minutes to complete.

### 2.4. Data Analysis

#### 2.4.1. Preprocessing

The EEG was preprocessed by a band-pass filter and further analyzed using EEGLAB [15] software in MatLab. The raw EEG was 1 to 50 Hz band-pass filtered. Each trace was visually inspected, and data predominated by electrical noise was discarded.

#### 2.4.2. Wavelet Transforms

Wavelet transform was used to transform a single-channel EEG signal into a time-frequency map. In this study, the continuous wavelet transform (CWT) was applied, and the Morlet wavelet was employed [16], and it is

(1) $$\psi_{0}\left(t\right)=\pi^{-1/4}e^{iwt}e^{-1/2t^{2}},$$

where *w* is the wavelet central angle frequency, often *w* ≥ 6, which is an optimal value to adjust the time-frequency resolution [17]. In this study, *w* = 6 was applied. Then, a family of wavelets can be generated: $\psi_{\text{s}}(t)=(1/\sqrt{2})\psi_{0}(t/s)$, s ∈ (0, +*∞*), and *s* is called a scale. The CWT at scale *s* and time *t* of a signal *x*(*t*) is defined as

(2) $$W\left(s,\tau \right)=\frac{1}{\sqrt{s}}\int x\left(t\right)\psi_{s}*\left(\frac{t-\tau }{s}\right)dt,$$

where *ψ*_*s*_ is a parent wavelet function, and ∗ denotes complex conjugation. By adjusting the scale *s* and the translation *τ*, a series of different frequency resolutions in the signal can be projected on the two-dimension space (scale *s* and translation *τ*). The factor $\sqrt{s}$ normalizes energy across the different scales.

#### 2.4.3. PARAFAC

**Figure 1:**
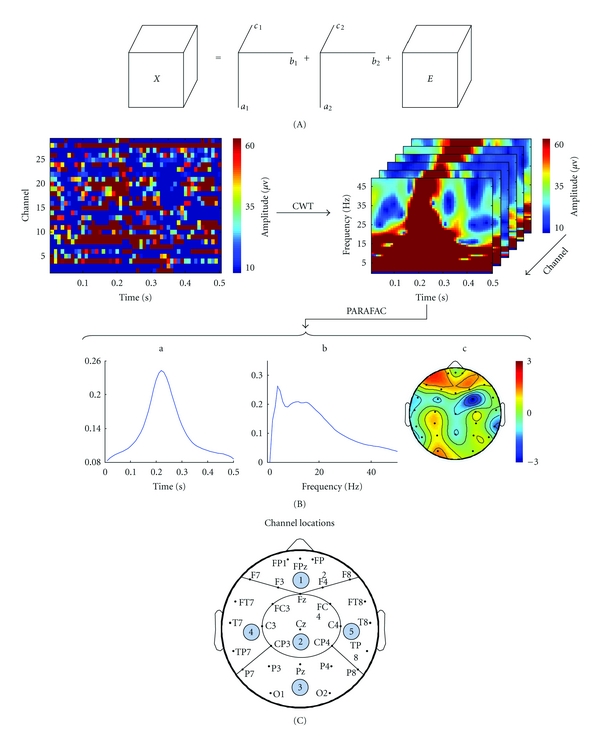
The PARAFAC model and factors extracted by the PARAFAC model from one case. (A) PARAFAC modeling of a three-way tensor. Each component (*R* = 2) is the outer product of *a*, *b*, and *c* of rank-1, and E is a residual tensor. (B) Flowchart describing the procedure of the epoch decomposition process by the PARAFAC method. (C) Whole brain cortex is clustered into 5 zones (1 = frontal, 2 = central, 3 = occipital, 4 = left temporal, and 5 = right temporal).

After wavelet transformation of all EEG channels, a three-way tensor *X*(*t*, *f*, *c*) (time-frequency-channel), giving the energy at time *t*, frequency *f*, and channel *c*, was obtained. To decompose the three-way tensor into time, frequency, and channel modes, the PARAFAC method was applied, and a linear combination of the three-way tensor was obtained by means of the alternating least squares (ALS) algorithm [14, 18]. The PARAFAC model is defined as

(3) $$X_{T\times F\times C}=\overset{\text{N}}{\underset{n=1}{\sum }}a_{n}\circ b_{n}\circ c_{n}+E,$$

where *N* is the number of signal factors of *X*_*T*×*F*×*C*_, and *a*_*n*_, *b*_*n*_, and *c*_*n*_ indicate the *n*th column of the loading matrices *A* ∈ *R* ^*T*×*N*^, *B* ∈ *R* ^*F*×*N*^, and *C* ∈ *R* ^*C*×*N*^, respectively. *A*, *B*, and *C* represent the time, frequency, and channel modes and provide information on the interactions between modes. *E* ∈ *R* ^*T*×*F*×*C*^ is the residual information in the decomposition. The operator ∘ represents the outer product of two vectors. Illustration of a 2-factor PARAFAC model on a three-way dataset is shown in Figure 1(A).

In the PARAFAC method, determination of the number of factors is a key issue. There are several methods to determine the number of factors, including the visual appearance of loadings, the residual analysis, the core consistency, and the number of iterations of the algorithm [19]. In this study, the core consistency method was employed. The core consistency represents the resemblance between the Tucker3 core and the PARAFAC core [14]. A Tucker3 model is similar to the PARAFAC model, and both of them are an extension of bilinear factor analysis to tensors [20]. The principle of the core consistency is as follows: (1) the PARAFAC model is available when the core consistency value is greater than 90%; (2) the PARAFAC model is not available when the core consistency value is less than 50%; (3) the PARAFAC model is probably available when the core consistency value is between 50 and 90%. The core consistency is defined as follows:

(4) $$\begin{array}{cc} & \text{Core-Consistency}\\ & \;\;\;=\left(1-\frac{\sum_{t=1}^{R}\sum_{j=1}^{R}\sum_{k=1}^{R}{\left(g_{ijk}-t_{ijk}\right)}^{2}}{R}\right)\times 100, \end{array}$$

where g_*ijk*_ and *t*_*ijk*_ are the Tucker3 core and the PARAFAC core, respectively; *R* is the factor number. In the PARAFAC core, *t*_*ijk*_ = 1 if *i* = *j* = *k*, otherwise *t*_*ijk*_ = 0, and in the Tucker3 core, *g*_*ijk*_ can be nonzero for all *i*, *j*, and *k*. This method for determining the factor number has been successfully applied to multiple neural data [13, 21].

#### 2.4.4. Statistics

Data were analysed in the time, frequency, and channel domains for all subjects. For the channel domain, the average energy for each stimulus was calculated for all channels and compared between the two groups, with outlier detection based on the generalized extreme studentized deviate (GESD) [22]. The average energy distribution for each group was obtained by averaging across all subjects. Cortical locations were grouped into 5 zones (frontal (FPz, FP1, FP2, Fz, F3, F4, F7, F8), central (Fz, Cz, FC3, FC4, C3, C4, CP3, CP4), occipital (Pz, CP3, CP4, P3, P4, P7, P8, O1, O2), left temporal (F7, F3, Fz, FT7, FC3, T7, C3, TP7, CP3, P7), and right temporal (F8, F4, Fz, FT8, FC4, T8, C4, TP8, CP4, P8)), as shown in Figure 1(C). The active ratios (average energy of one zone/the sum of energy across all zones) were assessed using the *t*-test. In time and frequency domains, the differences between pain and control groups were evaluated by *t*-test. 

The mean age of the chronic pain group (49 ± 11 years, *n* = 13) was significantly greater than the pain-free group (39 ± 11 years, *n* = 13) (*P* < 0.05, *t*-test). Multiple linear regression (MLR) was used to investigate the effect of age on the frequency domain parameter. The pain-free subjects and chronic pain subjects were represented by 0 and 1, respectively, and the effect of age and subject grouping differentiated by MLR as shown in Figure 4. In the MLR analysis, frequency was the dependent variable, while the age and subject grouping were independent variables. The effect of MLR was evaluated by the *F*-test.

## 3. Results

**Figure 2:**
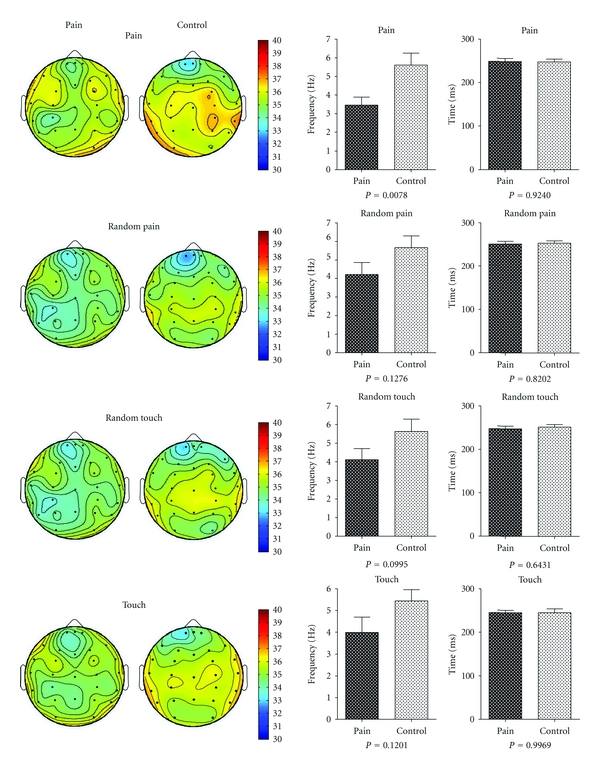
Results at the level of group corresponding to the four different stimuli. Left: the average energy distribution (pain, random pain, random touch, and touch, resp.). Middle: the statistical results in the frequency domain. Right: the statistical results in the time domain.

An example showing the epoch decomposition process using the PARAFAC method is illustrated in Figure 1(B). Wavelet decomposition was employed on the raw EEG recordings, generating time-frequency information corresponding to each channel. The frequency range for the wavelet decomposition was 1–50 Hz with an interval of 0.5 Hz. The factors were then extracted by the PARAFAC model from each epoch for every subject. Every factor was decomposed into three-way information at the time-frequency-channel domain. In the case shown (Figure 1(B) a, b, and c), the three-way information for one factor revealed a neural response frequency in the frontal region of 4 Hz and response time of 220 ms after the stimulus. Three-way information for each subject was obtained by averaging the results across all epochs. Comparisons between groups of the spatial topography, frequency, and time responses are shown in Figure 2.

### 3.1. Comparison in Frequency Domain

As shown in Figure 2 (middle), the frequency response in the chronic pain group (around 4 Hz) was lower than that in control group (around 5.5 Hz) and was statistically significant for the pain stimulus (chronic pain group: 3.456 ± 1.716 Hz; control group: 5.608 ± 2.315 Hz; *P* < 0.05, *t*-test).

### 3.2. Comparison in Time Domain

As shown in Figure 2 (right), the response time was around 250 ms in both groups and showed no significant difference for any of the stimuli.

### 3.3. Comparison in Location

**Figure 3:**
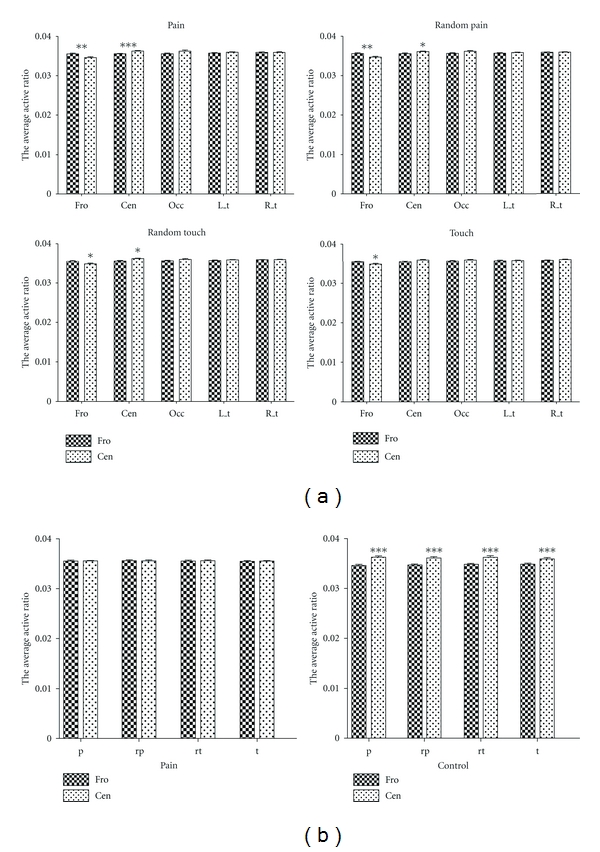
(a) Statistical results of the active ratio between the two groups corresponding to 5 zones under the four different stimuli. (b) Within-group comparison of the active ratio between the frontal and the central zones in the chronic pain group (left) and the corresponding result in the control group (right).

As shown in Figure 2 (left), the topographical analysis indicated that the active zone was mainly in the frontal region in pain group and in the central region in the control group. These differences were quantified by comparing the active ratios in the 5 cortical regions (see Figure 1(C)). The results are shown in Figure 3(a); in the frontal region, the active ratio of neural response in the chronic pain group was significantly higher than that in the control group for all 4 tests stimuli (chronic pain group: 0.03551 ± 0.00085, control group: 0.03459 ± 0.00080, pain:  *P* < 0.01; chronic pain group: 0.03557 ± 0.00088, control group: 0.03469 ± 0.00076, random pain:  *P* < 0.01; chronic pain group: 0.03551 ± 0.00077, control group: 0.03482 ± 0.00074, random touch:  *P* < 0.05; chronic pain group: 0.03545 ± 0.00061, control group: 0.03485 ± 0.00073, touch:  *P* < 0.05, *t-*test). In comparison, in the central region, the active ratio of neural response in the chronic pain group was lower than that in the control group and was statistically significant for all but the touch stimulus (chronic pain group: 0.03555 ± 0.00043, control group: 0.03624 ± 0.00051, pain:  *P* < 0.001; chronic pain group: 0.03557 ± 0.00077, control group: 0.03608 ± 0.00050, random pain:  *P* < 0.05; chronic pain group: 0.0356 ± 0.0006, control group: 0.03621 ± 0.00043, random touch:  *P* < 0.01; chronic pain group: 0.0355 ± 0.00042, control group: 0.03591 ± 0.00065, touch:  *P* = 0.0526, *t*-test). In other regions, there were no significant differences between the two groups.

Furthermore, we were interested in the differences in active ratio between the frontal and central regions within each group. As shown in Figure 3(b), in the pain group, the active ratios were not significantly different, whereas in the control group, the active ratios in the central region were significantly higher than in the frontal region for all stimuli (frontal: 0.03459 ± 0.00080, central: 0.03624 ± 0.00051, pain:  *P* < 0.0001; frontal: 0.03469 ± 0.00076, central: 0.03608 ± 0.00050, random pain:  *P* < 0.0001; frontal: 0.03482 ± 0.00074, central: 0.03621 ± 0.00043, random touch:  *P* < 0.0001; frontal: 0.03485 ± 0.00073, central: 0.03591 ± 0.00065, touch:  *P* < 0.001, *t*-test).

### 3.4. Effects of the Age on the Time Frequency and Active Ratio

**Figure 4:**
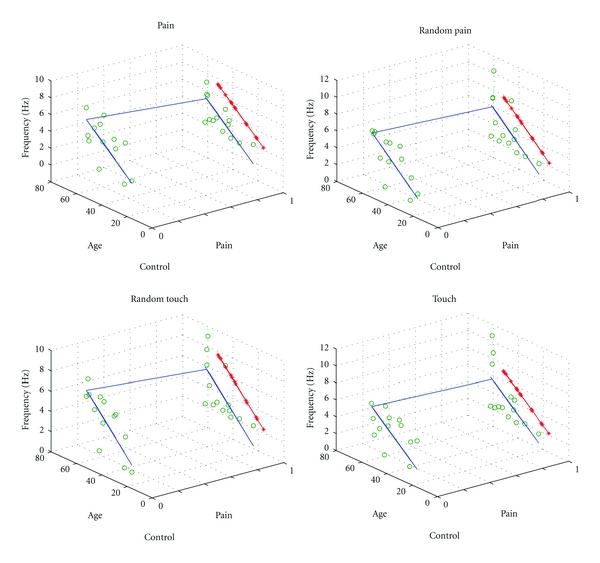
Influence of age and pain status on the frequency responses of subjects by MLR during the four different stimuli.

The mean age of the subjects in the pain group was significantly greater than in the control group. This study considered the effects of the age on the time, frequency, and active ratio parameters. Firstly, linear regression analysis was carried out on age versus response time, frequency, and active ratio; the only significant correlation was observed between age and response frequency. MLR was used to determine whether the difference in the frequency domain between two groups could be attributed to an age effect and is shown in Figure 4. The line marked by “∗” was the vertical mapping of the line of control group to the same plane with the line of the chronic pain group. From the comparisons of these lines, it is clear that the frequencies of the chronic pain group were significantly lower than those of the control group at the same age for the pain (*R* ^2^ = 0.4124, *F* = 24.131, *P* = 0.0017), random pain (*R* ^2^ = 0.3398, *F* = 6.177, *P* = 0.0069), random touch (*R* ^2^ = 0.4064, *F* = 8.2145, *P* = 0.0019), and touch (*R* ^2^ = 0.2156, *F* = 3.2985, *P* = 0.0542) stimuli. The differences in the frequency domain between the two groups therefore cannot be attributed entirely to the difference in age between groups.

## 4. Discussion

### 4.1. PARAFAC as an Effective Method for EEG Decomposition

In this study, the PARAFAC method was applied to somatosensory evoked potential recordings to analyze EEG time-frequency-channel domain [23] characteristics in chronic pain subjects. The advantage of this method is that it can extract more information in comparison with the two-way models (PCA and ICA) but also takes into account the frequency content of the signals in specific time periods across different channels [20]. The PARAFAC method has been successfully used to characterise the structure of epileptic seizure [14, 24, 25]. Analyzing the EEG signals in the time-frequency-channel domain, we found that the response latency was about 250 ms for all stimuli in both groups, while the frequency responses of the chronic pain group were lower than those of the control group. Furthermore, topographical analysis showed that the chronic pain group exhibited predominantly frontal cortical activity, compared to central activation in the control group. These findings are in accordance with previous results showing a response latency of about 250 ms [26], an increased size and reduction in population spike frequency [27], and restriction of some ERP components to frontal-central regions in patients with fibromyalgia syndrome [28]. Our findings indicate that the PARAFAC method is an effective technique for extracting the characteristics of multiple EEG recordings in the time-frequency-channel domain.

### 4.2. Activity Regions Involved in Chronic Pain

Previous studies have shown that the scale distribution of laser-evoked potentials (LEPs) around the chronic pain ERP components extends into vertex and frontocentral leads in fibromyalgia syndrome (FS) patients, indicating more widespread nociceptive activation outside the cortical hand area [28]. One question addressed in this study is whether the active ratio of frontal or central regions versus the whole cortex is different between the two groups. In this study, the active ratio was used to assess the activity within different regions of the cortex. The statistical results showed that the active ratios of the chronic pain group were significantly higher than those of the control group in the frontal region. The active ratios calculated from the other cortical regions showed no differences between the two groups. These results imply that evoked EEG activity in the frontal and central cortical regions may help discriminate between chronic pain and pain-free subjects. These findings are in agreement with the previous studies showing that some ERP components are more restricted to frontocentral regions in patients with fibromyalgia syndrome [28].

In keeping with the above findings, the active ratio in the central region was significantly higher than that in the frontal region in the control group, and vice versa for the chronic pain group. These results further indicate that the frontal cortical regions are involved in somatosensory processing in the chronic pain condition compared to central regions for pain-free subjects, in accordance with [29–32]. These findings suggest that passive functions (emotion, attention, etc.) are presented more frontally [33], and pain beliefs influence patients behavioral and psychological functioning because of their persistent pain experience [34].

### 4.3. Relationship between Frequency and Chronic Pain

In this study, we found that the cortical neural responses to somatosensory stimuli occurred around 250 ms in both of the groups and that the response frequency in the chronic pain group was lower than in the control group. In particular, the dominant activity was in the delta frequency range (around 4 Hz) for the chronic pain patients, compared to the theta frequency range (around 5.5 Hz) in the controls. Taken together, these results indicate that frontal cortical activation and a lower response frequency are characteristics of evoked EEG responses in chronic pain subjects.

In imaging studies, the functional connectivity between cortical structures receiving input arising from nociceptors has documented that experimental pain is processed in multiple pain-related areas, often characterized as a “pain network” [35, 36]. This “pain network” is not fixed but changes as a function of the pain-related task [37]. Moreover, in the study of cortical pathophysiology, pain-related neural networks are larger in patients with sympathetically mediated chronic pain (SMP) compared to acute pain states [32]. Therefore, the pain-related network of chronic pain patients is more extensive than that of the controls. This may be partly due to the effects of the patients' persistent pain experience, resulting in processing experimental pain accompanied with subjective experience. The larger pain-related network is accompanied with a lower frequency rhythm, which is consistent with data from isolated hippocampal slice experiments showing a reduction in the frequency of population spikes with increasing size of the participating neuronal population [27].

Theta activity is associated with alertness, attention, and the efficient processing of cognitive and perceptual tasks [38], while delta band activity is associated with pathological conditions associated with impairment of brain networks such as amnesic mild cognitive impairment (MCI) and Alzheimer's disease (AD) [39]. Chronic pain is also found to be frequently associated with psychiatric disorders [40]. Thus, the delta rhythm of chronic pain patients is suggestive of impairment to pain-related brain network processing.

### 4.4. Influence of Age on the Response Frequency

In this study, the mean age of the chronic pain group was significantly greater than that of the control group. MRL was applied to analyze the influences of age and pain status on the frequency responses of subjects. Our results showed that the frequency responses of the chronic pain group were significantly lower than those of the control group across all ages. Thus, the differences in the frequency domain between the two groups cannot be attributed entirely to the difference in age between groups.

In summary, the PARAFAC method with a continuous wavelet was used to extract time-frequency-channel domain information from somatosensory-evoked EEG recordings from chronic pain and pain-free subjects. We found that the response latency was about 250 ms, the chronic pain group had lower response frequency, and the central and frontal regions were the crucial regions of cortical activation. Further analysis indicated that the frontal regions were more involved in the chronic pain condition than the control condition. Application of MLR to the analysis of the relationship between frequency and age showed that the lower frequency response in the chronic pain group was not attributable to the difference in subject age. The conclusion from these findings is that the PARAFAC method is an effective tool for characterising multiple EEG recordings in the time-frequency-channel domain.

## References

[1] Niedermeyer E, da Silva FL (2004). *Electroencephalography: basic Principles, Clinical Applications, and Related Fields*.

[2] Ettinger AB, Argoff CE (2007). Use of antiepileptic drugs for nonepileptic conditions: psychiatric disorders and chronic pain. *Neurotherapeutics*.

[3] Zhuo M (2008). Cortical excitation and chronic pain. *Trends in Neurosciences*.

[4] de Pascalis V, Magurano MR, Bellusci A, Chen AC (2001). Somatosensory event-related potential and autonomic activity to varying pain reduction cognitive strategies in hypnosis. *Clinical Neurophysiology*.

[5] Sarnthein J, Jeanmonod D (2008). High thalamocortical theta coherence in patients with neurogenic pain. *NeuroImage*.

[6] Derbyshire SWG, Jones AKP, Creed F (2002). Cerebral responses to noxious thermal stimulation in chronic low back pain patients and normal controls. *NeuroImage*.

[7] Geha PY, Apkarian AV (2005). Brain imaging findings in neuropathic pain. *Current Pain and Headache Reports*.

[8] de Tommaso M, Libro G, Guido M, Sciruicchio V, Losito L, Puca F (2003). Heat pain thresholds and cerebral event-related potentials following painful CO2 laser stimulation in chronic tension-type headache. *Pain*.

[9] Stern J, Jeanmonod D, Sarnthein J (2006). Persistent EEG overactivation in the cortical pain matrix of neurogenic pain patients. *NeuroImage*.

[10] Valeriani M, Rambaud L, Mauguière F (1996). Scalp topography and dipolar source modelling of potentials evoked by CO_2_ laser stimulation of the hand. *Electroencephalography and Clinical Neurophysiology*.

[11] Bromm B, Lorenz J (1998). Neurophysiological evaluation of pain. *Electroencephalography and Clinical Neurophysiology*.

[12] Medina J, Coslett HB (2010). From maps to form to space: touch and the body schema. *Neuropsychologia*.

[13] Miwakeichi F, Martínez-Montes E, Valdés-Sosa PA, Nishiyama N, Mizuhara H, Yamaguchi Y (2004). Decomposing EEG data into space-time-frequency components using Parallel Factor Analysis. *NeuroImage*.

[14] Acar E, Aykut-Bingol C, Bingol H, Bro R, Yener B (2007). Multiway analysis of epilepsy tensors. *Bioinformatics*.

[15] Delorme A, Makeig S (2004). EEGLAB: an open source toolbox for analysis of single-trial EEG dynamics including independent component analysis. *Journal of Neuroscience Methods*.

[16] Li X, Yao X, Fox J, Jefferys JG (2007). Interaction dynamics of neuronal oscillations analysed using wavelet transforms. *Journal of Neuroscience Methods*.

[17] Farge M, Goirand E, Meyer Y, Pascal F, Wickerhauser MV (1992). Improved predictability of two-dimensional turbulent flows using wavelet packet compression. *Fluid Dynamics Research*.

[18] Smilde A, Bro R, Geladi P (2004). *Multi-Way Analysis with Applications in the Chemical Sciences*.

[19] Bro R, Kiers HAL (2003). A new efficient method for determining the number of components in PARAFAC models. *Journal of Chemometrics*.

[20] Acar E, Yener B (2009). Unsupervised multiway data analysis: a literature survey. *IEEE Transactions on Knowledge and Data Engineering*.

[21] Estienne F, Matthijs N, Massart DL, Ricoux P, Leibovici D (2001). Multi-way modelling of high-dimensionality electroencephalographic data. *Chemometrics and Intelligent Laboratory Systems*.

[22] Li X, Bowers CP, Schnier T (2010). Classification of energy consumption in buildings with outlier detection. *IEEE Transactions on Industrial Electronics*.

[23] Harshman RA (1970). Foundations of the PARAFAC procedure: models and conditions for an" explanatory" multi-modal factor analysis. *UCLA Working Papers in Phonetics*.

[24] de Vos M, Vergult A, de Lathauwer L (2007). Canonical decomposition of ictal scalp EEG reliably detects the seizure onset zone. *NeuroImage*.

[25] Acar E, Bingo CA, Bingo H, Yener B Computational analysis of epileptic focus localization.

[26] Pazzaglia C, Valeriani M (2009). Brain-evoked potentials as a tool for diagnosing neuropathic pain. *Expert Review of Neurotherapeutics*.

[27] Fox JE, Bikson M, Jefferys JG (2007). The effect of neuronal population size on the development of epileptiform discharges in the low calcium model of epilepsy. *Neuroscience Letters*.

[28] Lorenz J, Grasedyck K, Bromm B (1996). Middle and long latency somatosensory evoked potentials after painful laser stimulation in patients with fibromyalgia syndrome. *Electroencephalography and Clinical Neurophysiology*.

[29] Casey KL, Lorenz J, Minoshima S (2003). Insights into the pathophysiology of neuropathic pain through functional brain imaging. *Experimental Neurology*.

[30] Neugebauer V, Galhardo V, Maione S, Mackey SC (2009). Forebrain pain mechanisms. *Brain Research Reviews*.

[31] Moisset X, Bouhassira D (2007). Brain imaging of neuropathic pain. *NeuroImage*.

[32] Apkarian AV, Thomas PS, Krauss BR, Szeverenyi NM (2001). Prefrontal cortical hyperactivity in patients with sympathetically mediated chronic pain. *Neuroscience Letters*.

[33] Picard N, Strick PL (1996). Motor areas of the medial wall: a review of their location and functional activation. *Cerebral Cortex*.

[34] Jensen MP, Romano JM, Turner JA, Good AB, Wald LH (1999). Patient beliefs predict patient functioning: further support for a cognitive-behavioural model of chronic pain. *Pain*.

[35] Rainville P, Carrier B, Hofbauer RK, Bushnell MC, Duncan GH (1999). Dissociation of sensory and affective dimensions of pain using hypnotic modulation. *Pain*.

[36] Davis JL (2000). Use of sibutramine hydrochloride monohydrate in the treatment of the painful peripheral neuropathy of diabetes. *Diabetes Care*.

[37] Ohara S, Crone NE, Weiss N, Lenz FA (2006). Analysis of synchrony demonstrates "pain networks" defined by rapidly switching, task-specific, functional connectivity between pain-related cortical structures. *Pain*.

[38] Stern RM, Ray WJ, Quigley KS (2001). *Psychophysiological Recording*.

[39] Babiloni C, Frisoni G, Vecchio F (2010). Global functional coupling of resting EEG rhythms is abnormal in mild cognitive impairment and Alzheimer's disease: a multicenter EEG study. *Journal of Psychophysiology*.

[40] Dersh J, Polatin PB, Gatchel RJ (2002). Chronic pain and psychopathology: research findings and theoretical considerations. *Psychosomatic Medicine*.

